# Incidence patterns of rare cancer in southeast Asian and western Pacific countries (RARECAREnet Asia project): a study using population-based cancer registry data, 2011–2015

**DOI:** 10.1016/j.lansea.2025.100670

**Published:** 2025-09-12

**Authors:** Patumrat Sripan, Siti Norbayah Yusof, Donsuk Pongnikorn, Imjai Chitapanarux, Balqis Bahtiar, Nor Saleha Ibrahim Tamin, Karnchana Daoprasert, Narate Waisri, Young-Joo Won, RuRu Chun-Ju Chiang, Annalisa Trama, Hadrien Charvat, Kriengkrai Srithanaviboonchai, Tomohiro Matsuda

**Affiliations:** a Research Institute for Health Sciences, Chiang Mai University, Chiang Mai, Thailand; b National Cancer Institute, Putrajaya, Malaysia; c Department of Medical Services, Ministry of Public Health, Nonthaburi, Thailand; d Chiang Mai Cancer Registry, Faculty of Medicine, Chiang Mai University, Chiang Mai, Thailand; e Division of Radiation Oncology, Department of Radiology, Faculty of Medicine, Chiang Mai University, Chiang Mai, Thailand; f Ministry of Health, Putrajaya, Malaysia; g Cancer Registry Unit, Lampang Cancer Hospital, Lampang, Thailand; h Division of Health Administration, Yonsei University, Wonju, Republic of Korea; i Taiwan Cancer Registry Center, Institute of Epidemiology and Preventive Medicine College of Public Health, National Taiwan University, Taipei, Taiwan; j Research Department, Fondazione IRCSS, Istituto Nazionale dei Tumouri, Milan, Italy; k Faculty of International Liberal Arts, Juntendo University, Tokyo, Japan; l Division of International Health Policy Research, Institute for Cancer Control, National Cancer Center, Tokyo, Japan

**Keywords:** Rare cancer, Southeast Asia, East Asia, Population-based cancer registry

## Abstract

**Background:**

In Southeast Asia (SEA), the understanding of most rare cancers is limited, which sometimes leads to delays in diagnosis, treatment, and care. This study aimed to estimate for the first time the incidence of rare cancers in SEA using population-based cancer registry (PBCR) data from the 2011–2015 period.

**Methods:**

This study used data from the nationwide PBCR of Malaysia and two PBCRs in northern Thailand in Chiang Mai and Lampang Cancer registries. The age-standardized incidence rate (ASR) per 100,000 person-year of the rare cancers included in the RARECAREnet list was calculated. All analyses were performed using SEER∗Stat (version 8.3.5). Cancers defined as rare by RARECAREnet in Europe were also rare in Thailand and Malaysia.

**Findings:**

The ASR of some rare cancers in Thailand and Malaysia were greater than that in Japan, Korea, and Taiwan, including some pediatric cancers (pancreatoblastoma and odontogenic malignant tumors) in Malaysia, eye and adnexal cancer, and epithelial tumors of the penis in Thailand. ASR of nasopharyngeal cancer was higher in Thailand and Malaysia than in Japan and Korea but lower compared to Taiwan.

**Interpretation:**

Although most rare cancers were also rare in Thailand and Malaysia, some cancers were not considered rare. However, the incidence of some rare cancers in Thailand and Malaysia were higher than that in Japan, Korea, and Taiwan. To enhance understanding, diagnosis, treatment, and care of rare cancers, reliable epidemiological data needs to be generated under the RARECAREnet Asia project by working with countries in Asia with high-quality PBCRs.

**Funding:**

This study was supported by a UICC Yamagiwa-Yoshida Memorial International Cancer Study Grant (Award/Grant Number: YY/2022-1477) and Government of Japan Ministry of Health Labour and Welfare Grant numbers: 23EA1033, and was partially supported by 10.13039/501100002842Chiang Mai University, Thailand.

**Research in context**

**Evidence before this study**

There is a critical need for Asian countries—which account for over 40% of global cancer diagnoses—to develop and implement cancer control strategies based on robust, population-based cancer registry (PBCR) data. An internationally agreed upon definition of rare cancers was developed in Europe. Thus, the Surveillance of Rare Cancers in Asia (RARECAREnet Asia) project was established by the National Cancer Center, Japan, National Cancer Center, Korea, and Taiwan Cancer Registry Center in collaboration with the Fondazione IRCCS Istituto Nazionale Tumouri, Milan (INT), to measure the burden of rare cancers in Asia. The first RARECAREnet Asia study showed that most rare cancers in Europe were also rare in Asian countries. The observed differences were attributed to well-known risk factors. The European definition and list of rare cancers seem to accurately reflect the same cancer incidence in East Asia. The initial findings from RARECAREnet Asia indicated that most cancers classified as rare in Europe are also rare in Asian populations, with observed differences largely attributable to established risk factors. The European definition and classification of rare cancers appear to be applicable to East Asian contexts. Furthermore, when an alternative definition was used in an epidemiological study of rare cancers in India and other South Asian countries, the study found that oral cavity cancers are considered rare in Europe, with crude incidence rates (CR) of ≤6 per 100,000 population, whereas cancers of the pancreas, rectum, urinary bladder, and melanomas are more prevalent. Additionally, uterine, colon, and prostate cancers are classified as rare in India, Nepal, and Bhutan.

**Added value of this study**

This study provides the first estimates of rare cancer incidence in Southeast Asia, utilizing data from population-based cancer registries in Malaysia and Thailand. The majority of rare cancers identified in the RARECAREnet classification were also found to be rare within Southeast Asia. However, the age-standardized rates (ASR) for certain rare cancers in this region, including specific pediatric cancers such as pancreatoblastoma and odontogenic malignant tumors in Malaysia, as well as eye and adnexal cancers and epithelial tumors of the penis in Thailand, were higher than those reported in East Asia. ASR of nasopharyngeal was highest in Taiwan while it is higher in SEA than Japan and Korea. Our findings provide a foundation for analyzing incidence trends of specific cancers, particularly infection-related rare cancers that are more prevalent in Thailand and Malaysia than in East Asia.

**Implications of all the available evidence**

The evaluation of incidence trends for rare cancers was constrained by the limited availability of high-quality longitudinal data. The trend in incidence of rare cancers in Japan has been reported but this has not yet been documented in SEA. Comprehensive analysis over a ten-year period is essential to accurately detect increases in rare cancer incidence and to inform the prioritization of public health interventions, especially for infection-related rare cancers that are more common in Thailand and Malaysia compared to East Asia. Expanding this research to include South Asian countries would further enrich the existing body of evidence on rare cancers in Asia.

## Introduction

There is an overwhelming need for Asian countries, where more than 40% of current cancer diagnoses occur, to adopt and implement cancer control strategies informed by reliable population-based cancer registry (PBCR) data.^1^ In East Asian countries including Japan, Korea, and Taiwan, the distribution of primary cancer sites was estimated to differ across regions^2^ with regard to both common and rare cancers. An internationally agreed upon definition of rare cancer was developed in Europe.^3^ Thus, the Surveillance of Rare Cancers in Asia (RARECAREnet Asia) project was established by the National Cancer Center, Japan, National Cancer Center, Korea, and Taiwan Cancer Registry Center in collaboration with the Fondazione IRCCS Istituto Nazionale Tumouri, Milan (INT), to measure the burden of rare cancers in Asia. The first RARECAREnet Asia study showed that most rare cancers in Europe were also rare in Asian countries. The observed differences were attributed to well-known risk factors.^2^ The European definition and list of rare cancers seem to accurately reflect the same cancer incidence in East Asia. Furthermore, applying an alternative definition of rare cancer, an epidemiological study of rare cancers in India and other South Asian countries found that oral cavity cancers are considered rare in Europe, with crude incidence rates (CR) of ≤6 per 100,000 population, whereas cancers of the pancreas, rectum, urinary bladder, and melanomas are more prevalent. Additionally, uterine, colon, and prostate cancers are classified as rare in India, Nepal, and Bhutan.^4^ The RARECAREnet Asia publications were limited to Japan, Taiwan and Korea; thus, the incidence pattern of rare cancers in Southeast Asia (SEA) is limited. This study aimed to estimate, for the first time, the incidence of rare cancers in Thailand and Malaysia and compare the results to those of Japan, Korea, and Taiwan using population-based cancer registry (PBCR) data.

## Methods

The incident data from multiple fields that are as patient demographics, diagnoses and tumor characteristics as commonly collected by PBCRs.^5^ As part of the RARECAREnet Asia project, this study analyzed population-based cancer registry (PBCR) data from 2011 to 2015. In Thailand, data from two long-standing PBCRs in northern Thailand, the Chiang Mai Cancer Registry and the Lampang Cancer Registry, covering 25% of the Northern Thai population (6,350,499), were included. In Malaysia, the PBCR has had nationwide coverage since 2007 (34,308,530). While, the PBCR in Japan has covered 100% of the population since 2012. However, in the 2011–2015 period, high-quality prefectural data (DCO% <10%) were available for 37 to 43 prefectures out of the 47 prefectures, which corresponded to 446,045,783 people. In Korea and Taiwan, the coverage has been 100%. The population for the five-year period was 252,730,670 in Korea and 116,675,104 in Taiwan.

Systematic data checks were performed to detect errors, inconsistencies, or unusual combinations of site, morphology, sex, and age at diagnosis according to IARC/IACR check logic.^6^ A standard list of rare cancers was provided by the RARECAREnet expert panel and endorsed by major European cancer organizations.^7^ The following data quality indicators were calculated for the incidence of malignant cancers, the latter 2 of which are rare cancer specific.

1) proportion of cases known from death certificate only (DCO)
2) proportion of cases diagnosed incidentally at autopsy
3) proportion of Microscopically Verified (MV) cases
4) proportion of Not Otherwise Specified (NOS) morphology (for solid cancers: ICD-O-3 8000 Neoplasm, malignant; 8001 Tumor cells, malignant; 8010 Carcinoma, NOS; for hematological diseases: ICDO3 9590 Lymphoma, NOS; 9591 Non-Hodgkin lymphoma, NOS; 9760 Immunoproliferative diseases, NOS; 9820 Lymphoid leukemia, NOS; 9800 Leukemia, NOS; 9801 Acute leukemia, NOS 9860 Myeloid leukemia, NOS; and 9989 Myelodysplastic syndromes, NOS)
5) proportion of NOS topography (ICD-O-3: C14.0; C14.8; C26.0; C26.8; C26.9; C39.0; C39.8-C39.9; C57.7; C57.8; C57.9; C55.9; C63.2; C63.8; C63.9; C68.8; C68.9; C75.2; C75.4; C75.5; C75.8; C75.9; C76.0–76.8)

### List of cancers and definition of rare cancers

The standard list of rare cancers was provided by RARECAREnet expert panel and endorsed by major European cancer organizations.^8^ The list is organized into three tiers: the bottom tier (Tier 3) corresponds to the World Health Organization (WHO) names of individual cancer entities (http://whobluebooks.iarc.frn/) and their corresponding ICD-O-3 codes. The Tier 3 entities were grouped into categories (Tier 2) according to their morphologies and topographies. These entities must be viewed as clinically relevant by clinicians and correspond to consistent diagnostic and therapeutic approaches. The Tier 2 entities were then assembled into Tier 1 entities, which also included the NOS morphologies of any site. Tier 1 entities were intended to be major cancer entities in a clinical sense and to have organizational importance; for example, they could underline patient referral policies. However, focusing on the referral of patients, Tier 1 entities have been grouped into gross partitions called “Families”, identifying major groups of cancer diseases. Accordingly, the 12 families of rare cancers are as follows: 1. head and neck, 2. digestive rare, 3. thoracic rare, 4. Female genital rare, 5. male genital and urogenital rare, 6. skin rare, 7. pediatric, 8. sarcomas, 9. neuroendocrine, 10. endocrine organ, 11. central nervous system, and 12. hematological. Likewise. Similarly, the 6 families of common cancers are 1) digestive common, 2) female genital common, 3) thoracic common, 4) breast, 5) male genital and urogenital common, and 6) skin common.^8^

### Statistical analysis

Data were derived from cancer patients diagnosed between 2011 and 2015 from two long-standing PBCRs in northern Thailand in the Chiang Mai Cancer Registry and Lampang Cancer Registry and from the nationwide cancer registry in Malaysia. To check the applicability of the RARECAREnet list to Asian data, the age-standardized rate (ASR) was calculated according to the total person-years in the covered area in the general population of both sexes and adjusted by the Segi's world standard population to compare the incidence risks of rare cancers in Asian countries using the SEER∗Stat. Using R programs,^9^ the rate ratios (RRs) were calculated to compare the incidence of cancers between SEA and East Asia, Thailand and each of the East Asian countries (Japan, Korea, and Taiwan) and Malayasia and each of these countries, and the 95% confidence intervals (CIs) were also estimated. Only the aggregate results were shared and compared with those in Japan, Korea, and Taiwan on the basis of a previous publication.^2^

### Ethical statement

This study was approved by the Research Ethics Committee of the Lampang Cancer Hospital and Ministry of Health of Malaysia. The ethical approval for the data from Japan, Korea, and Taiwan was not necessary since the comparison was conducted based on the previous publication. The need for consent to participate was waived by ethic committees since the secondary data from PBCR that information is not individually identifiable were used.

This study used REPCAN (Guideline for REporting Population-based CANcer Registry Data) for analysis and reporting data.^10^

## Results

The proportion of microscopically verified (MV%) cases was satisfactory for most cancer sites except for the pancreas, liver and intrahepatic bile tract (IBT), gallbladder and extrahepatic biliary tract, and lung cancers. In Thailand, the MV% by cancer site ranged from 18% to 100%. The MV% was lower than 80% in some cancer sites, including epithelial tumors of the liver and intrahepatic bile tract (18%), epithelial tumors of the pancreas (35%), epithelial tumors of gallbladder (50%) and extrahepatic biliary tract (EBT) (53%), epithelial tumors of lung (57%), carcinomas of adrenal cortex (73%), and epithelial tumors of kidney (74%). In Malaysia, the MV% by cancer site ranged from 73% to 100%. The MV% was lower than 80% only for epithelial tumors of the liver and IBT (73%) (Supplementary Table S1).

Most rare cancers in the EU were also rare in northern Thailand and Malaysia. The ASR of several head and neck cancers, including oropharynx, eye and adnexa; digestive rare cancers, including gallbladder and extrahepatic biliary tract cancers; female genital rare cancers, including epithelial tumors of the vulva and vagina; male genital and urogenital rare cancers, including epithelial tumors of the penis; skin rare cancers, including Kaposi's sarcoma; sarcomas, including soft tissue sarcoma; neuroendocrine tumors; and central nervous system and hematological cancers, including lymphoid diseases, was significantly greater in northern Thailand than in Malaysia. For all common cancers, the ASR in Thailand was greater than that in Malaysia. Most of the cancers in the RARECAREnet list had lower ASRs in Malaysia than in Thailand, except for the nasopharynx in the head and neck cancer family; pancreatoblastoma, olfactory neuroblastoma, and odontogenic malignant tumor in the pediatric cancer family; and carcinoma of the pituitary gland in the endocrine cancer family (Table 1).

**Table 1:** Rare cancers in Thailand and Malaysia (RARECAREnet list).

Cancer site	Thailand			Malaysia		
	Count	ASR	95% CI	Count	ASR	95% CI
1. Head and Neck cancer family						
Epithelial tumors of Nasal Cavity and Sinuses	43	0.23	0.16–0.30	276	0.19	0.17–0.21
Epithelial tumors of Nasopharynx	341	1.93	1.73–2.13	4478	3.04	2.95–3.13
Epithelial tumors of Major Salivry Glands and Salivary Gland	129	0.72	0.60–0.84	825	0.57	0.53–0.61
Epithelial tumors of Hypopharynx and Larynx	255	1.23	1.08–1.38	1071	0.79	0.74–0.84
Epithelial tumors of Oropharynx	184	0.92	0.79–1.05	535	0.38	0.35–0.41
Epithelial tumors of Oral Cavity and Lip	413	2.02	1.83–2.21	1636	1.17	1.11–1.23
Epithelial tumors of Eye and Adnexa	25	0.14	0.09–0.19	46	0.03	0.02–0.04
Epithelial tumors of Middle Ear	1	0.01	0.00–0.03	34	0.02	0.01–0.03
2. Digestive Rare Family						
Epithelial tumors of Small Intestine	35	0.18	0.12–0.24	222	0.16	0.14–0.18
Epithelial tumors of Gallbladder and Extrahepatic Biliary Tract	506	2.33	2.13–2.53	1044	0.76	0.71–0.81
Epithelial tumors of Anal Canal	67	0.35	0.27–0.43	255	0.19	0.17–0.21
3. Thoracic Rare						
Epithelial tumors of Trachea	5	0.03	0.00–0.06	16	0.01	0.01–0.01
Epithelial tumors of Thymus	7	0.04	0.01–0.07	107	0.07	0.06–0.08
Malignant Mesothelioma	5	0.03	0.00–0.06	45	0.03	0.02–0.04
4. Female Genital Rare						
Non-Epithelial Tumors of Ovary	36	0.31	0.21–0.41	333	0.21	0.19–0.23
Epithelial Tumors of Vulva and Vagina	72	0.36	0.28–0.44	224	0.16	0.14–0.18
Trophoblastic Tumors of Placenta	9	0.07	0.02–0.12	77	0.05	0.04–0.06
5. Male Genital and Urogenital						
Testicular and Paratesticular	31	0.26	0.17–0.35	493	0.30	0.27–0.33
Epithelial Tumors of Penis	104	0.56	0.45–0.67	115	0.08	0.07–0.09
Epithelial Tumors of Pevis and Ureter	111	0.53	0.43–0.63	140	0.11	0.09–0.13
Extragonadal Germ Cell Tumors	28	0.27	0.17–0.37	236	0.15	0.13–0.17
Epithelial Tumors of Urethra	12	0.06	0.03–0.09	11	0.01	0.00–0.02
6. Skin Rare						
Malignant Melanoma of Mucosa and Extracutaneous	8	0.04	0.01–0.07	25	0.02	0.01–0.03
Malignant Melanoma of Uvea	5	0.03	0.00–0.06	3	0.00	0.00–0.00
Adenexal Carcinomas of Skin	25	0.12	0.07–0.17	156	0.11	0.09–0.13
Kaposi's Sarcoma	20	0.16	0.09–0.23	35	0.02	0.01–0.03
7. Pediatric						
Neuroblastoma and Ganglioneuroblastoma	8	0.18	0.06–0.30	69	0.06	0.05–0.07
Nephroblastoma	7	0.17	0.04–0.30	59	0.05	0.04–0.06
Embryonal Tumors of Eye	12	0.30	0.13–0.47	104	0.09	0.07–0.11
Hepatoblastoma	3	0.07	0.00–0.15	53	0.05	0.04–0.06
Pleuropulmonary Blastoma	0	0.00	0.00–0.00	2	≈0.00	0.00–0.00
Pancreatoblastoma	0	0.00	0.00–0.00	25	0.02	0.01–0.03
Olfactory Neuroblastoma	3	0.01	0.00–0.02	30	0.02	0.01–0.03
Odontogenic Malignant Tumor	1	0.01	0.00–0.03	36	0.02	0.01–0.03
8. Sarcoma						
Soft Tissue Sarcoma	312	2.02	1.80–2.24	1943	1.32	1.26–1.38
Bone Sarcoma	74	0.60	0.46–0.74	749	0.49	0.45–0.53
Gastrointestinal Stromal Sarcoma	57	0.30	0.22–0.38	311	0.22	0.20–0.24
9. Neuroendocrine						
Net GEP	65	0.39	0.30–0.48	230	0.16	0.14–0.18
Net Lung	9	0.04	0.01–0.07	19	0.01	0.01–0.01
Net Other Sites	84	0.45	0.35–0.55	227	0.16	0.14–0.18
10. Endocrine						
Carcinomas of Pituitary Gland	1	0.00	0.00–0.00	63	0.04	0.03–0.05
Carcinomas of Thyroid Gland	597	3.78	3.48–4.08	2664	1.79	1.72–1.86
Carcinomas of Parathyroid Gland	5	0.04	0.00–0.08	12	0.01	0.00–0.02
Carcinomas of Adrenal Cortex	11	0.07	0.03–0.11	55	0.04	0.03–0.05
11. Central Nervous System (CNS)						
Central Nervous System (CNS)	306	1.93	1.71–2.15	1675	1.15	1.09–1.21
Embryonal Tumors of CNS	18	0.30	0.16–0.44	258	0.20	0.18–0.22
12. Hematological Rare						
Lymphoid Disease	1646	9.87	9.39–10.35	7948	5.51	5.39–5.63
Acute Myeloid Leukemia and Related Precursor Neoplasms	366	2.53	2.27–2.79	2256	1.56	1.50–1.62
Myeloid and Lymphoid Neoplasms	21	0.12	0.07–0.17	142	0.10	0.08–0.12
Myelopholiferative Neoplasms	109	0.70	0.57–0.83	581	0.38	0.35–0.41
Myelodysplastic Syndrome and Myelodysplastic/Myelopholiferative Diseases	11	0.05	0.02–0.08	30	0.02	0.01–0.03
Histiocytic and Denritic Cell Neoplasms	5	0.10	0.01–0.19	10	0.01	0.00–0.02
13. Digestive Common						
Epithelial Tumors of Esophagus	217	1.10	0.95–1.25	1120	0.82	0.77–0.87
Epithelial Tumors of Stomach	935	4.64	4.34–4.94	2879	2.09	2.01–2.17
Epithelial Tumors of Colon (including appendix)	1896	9.38	8.96–9.80	9719	7.04	6.90–7.18
Epithelial Tumors of Rectum	1070	5.42	5.10–5.74	4724	3.41	3.31–3.51
Epithelial Tumors of Pancreas	509	2.46	2.25–2.67	1903	1.40	1.34–1.46
Epithelial Tumors of Liver and Intrahepatic Bile Tract (IBT)	4703	23.51	22.84–24.18	3996	2.89	2.80–2.98
14. Thoracic Common						
Epithelial Tumors of Lung	5395	25.67	24.99–26.35	10,938	8.02	7.87–8.17
15. Female Genital Common						
Epithelial Tumors of Corpus Uteri	496	2.53	2.31–2.75	2523	1.75	1.68–1.82
Epithelial Tumors of Cervix Uteri	1238	7.04	6.65–7.43	3985	2.74	2.65–2.83
Epithelial Tumors of Ovary and Fallopian Tube	454	2.40	2.18–2.62	3186	2.18	2.10–2.26
16. Male Genital Common						
Epithelial Tumors of Prostate	766	3.40	3.16–3.64	3948	3.05	2.95–3.15
Epithelial Tumors of Kidney	242	1.24	1.08–1.40	1625	1.17	1.11–1.23
Epithelial Tumors of Bladder	672	3.11	2.87–3.35	2047	1.51	1.44–1.58
17. Skin Common						
Malignant Skin Melanoma	96	0.52	0.42–0.62	312	0.22	0.20–0.24
Epithelial Tumors of Skin	874	3.79	3.54–4.04	2620	1.93	1.86–2.00
18. Epithelial Tumors of Breast	3240	17.64	17.03–18.25	20,823	14.29	14.10–14.48

**Fig. 1:**
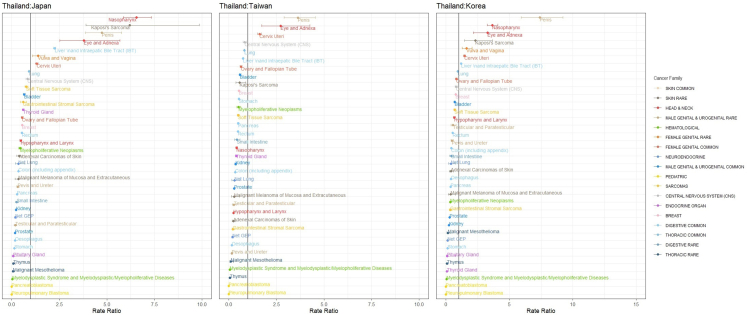
The rate ratios of the different types of cancer based on Tier 1 rare cancer classification between Thailand and East Asian countries, including Japan, Korea, and Taiwan. Only cancer sites that the rate is significantly lower (95% CI of RR < 1) or higher (95% CI of RR > 1). Only cancer sites that the rate is significantly lower (95% CI of RR < 1) or higher (95% CI of RR > 1) in Thailand compared to Japan, Korea, and Taiwan are shown. The color code indicates the family of cancer.

The incidence of most rare cancers was as low as that found in East Asian countries, namely, Japan, Korea, and Taiwan. However, some differences in incidence rates were observed. Fig. 1 shows the rate ratios of the different types of cancer based on Tier 1 classification between Thailand and each of the East Asian countries (Japan, Korea, and Taiwan).

**Fig. 2:**
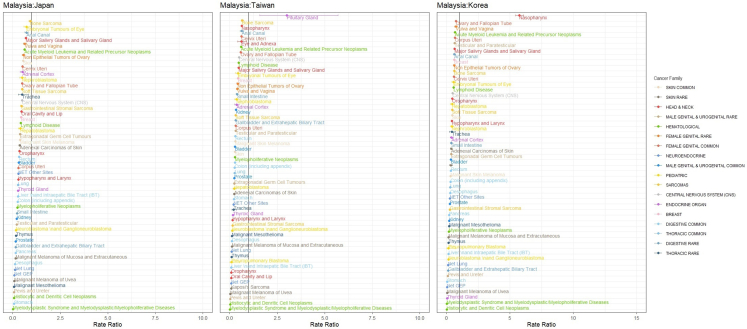
The rate ratios of the different types of cancer based on Tier 1 rare cancer classification between Malaysia and East Asian countries, including Japan, Korea, and Taiwan. Only cancer sites that the rate is significantly lower (95% CI of RR < 1) or higher (95% CI of RR > 1) Only cancer sites that the rate is significantly lower (95% CI of RR < 1) or higher (95% CI of RR > 1) in Malaysia compared to Japan, Korea, and Taiwan are shown. The color code indicates the family of cancer.

Fig. 2 shows the rate ratios of different types of cancer based on Tier 1 classification between Malaysia and Japan, Korea and Taiwan. The rate ratio was lower than 1 for most cancers, particularly for some pediatric cancers, such as pleuropulmonary blastoma, embryonal tumors of the eye, nephroblastoma, neuroblastoma and ganglioneuroblastoma; hematological rare cancers, including histiocytic and dendritic cell neoplasms; and myelodysplastic syndrome and myelodysplastic/myeloproliferative diseases.

## Discussion

This study represents the first estimation of rare cancer incidence in SEA using data from PBCRs in Malaysia and Thailand. Most of the rare cancers listed in the RARECAREnet list were also rare in Thailand and Malaysia. However, the ASR of some rare cancers in Thailand and Malaysia are higher than that in Japan, Korea, and Taiwan including some pediatric cancers (pancreatoblastoma and odontogenic malignant tumors) in Malaysia; eye and adnexal cancers and epithelial tumors of the penis are common in Thailand. ASR of nasopharyngeal was highest in Taiwan while it is higher in Thailand and Malaysia than Japan and Korea.

Most cancers within the 12 families were classified as rare and included eye and adnexa, gallbladder and extrahepatic biliary tract, epithelial tumors of vulva and vagina, epithelial tumors of penis, Kaposi's sarcoma, soft tissue sarcoma, nonmetastatic neuroendocrine tumor (NET), central nervous system tumors, and lymphoid diseases; these cancers exhibited significantly greater incidence rates in northern Thailand than in Malaysia. However, the incidence of epithelial tumors of nasopharynx, pancreatoblastoma, olfactory neuroblastoma, odontogenic malignant tumor, and carcinoma of the pituitary gland was greater in Malaysia than in Thailand.

In our study, nasopharyngeal cancer was rare in Thailand and Malaysia, with lower incidence rates observed in Thailand than in Malaysia. These results are consistent with those of a published study in which Indonesia, Vietnam, Singapore, Malaysia, and Brunei were the five countries in SEA with the highest ASR of nasopharyngeal cancer in Asia.^11^ Whereas, our study revealed that the incidence of epithelial tumors of the nasopharynx in Thailand was greater than that in Japan and Korea, except for Taiwan. Factors such as a history of cigarette smoking, chronic ear or nose disease, occupational exposure to wood dust, and lower education were associated with an increased risk of nasopharyngeal cancer in the Thai population.^12^ In Malaysia, nasopharyngeal carcinoma ranks eighth in prevalence, constituting 3.1% of all cases^13^ according to the National Cancer Registry database spanning 2017–2021. The highest incidences are observed among Chinese Malaysian males (9.0 per 100,000) and other indigenous Malaysian males (8.9 per 100,000), compared to national rates of 5.1 per 100,000 for all men and 1.7 per 100,000 for all women in Malaysia. Chinese Malaysians represent the second-largest ethnic group, comprising approximately 23% of Malaysia's population^14^; these people are largely descendants of southern Chinese immigrants who settled between the early 19th and mid-20th centuries. Moreover, genetic factors play a significant role in the pathophysiology of nasopharyngeal cancer, which has a markedly greater incidence in South China, where the incidence is 20–50 times greater than that in Western regions.^15^ Among the indigenous groups in Malaysia, the Bidayuh community in East Malaysia has a notably heightened risk of nasopharyngeal carcinoma.^16^ In addition to genetic predispositions, risk factors associated with the development of this cancer in Malaysia include family history, tobacco use, consumption of salted fish, exposure to domestic wood cooking fires, and occupational exposure to solvents and wood dust.^16^^,^^17^ In 2022, mortality rates of nasopharyngeal cancer exhibited substantial disparities, with age-standardized mortality rates in Indonesia, Malaysia, and Brunei reaching up to thirty times those reported in the US. Furthermore, the mortality-to-incidence ratio was lowest in Singapore and Thailand, highlighting the influence of socioeconomic factors on survival outcomes.^18^

One of the factors associated with nasopharyngeal cancer in Thailand is Epstein–Barr virus (EBV). Interestingly, most of the cancers, both rare and common, were found to be significantly higher in Thailand than in Japan, Korea, and Taiwan were infection-related cancer, including nasopharyngeal cancer caused by EBV, Kaposi's sarcoma caused by HIV, epithelial tumors of penis cancer, epithelial tumors of the vulva and vagina caused by HPV, and common cancers, including epithelial tumors of cervical cancer caused by HPV and epithelial tumors of liver and intrahepatic bile tract caused by hepatocellular carcinoma (HBV) and *Opisthorchisviverrini* (OV). Most of these infections, which can cause both common and rare forms of cancer, are sexually transmitted infections (STIs) that typically spread through unprotected sexual contact. While infection-related cancers may be caused by bacteria, viruses, or parasites, not every case involves an STI. The virus can also spread through bodily fluids such as blood or semen but is most often transmitted via saliva.

Thailand has robust programs to prevent STIs, which include the prevention of HIV and HPV. The prophylactic quadrivalent HPV vaccine, which can prevent cervical cancer, is likely cost-effective in the Thai population.^19^ However, the coverage of HPV vaccination remains low.^20^ A study in the Asian population revealed a greater incidence of HPV-attributable cancers in Thailand than in Japan and Korea, with rates of 64.6% in Thailand, 51.8% in Japan and 61.0% in Korea.^21^ This finding aligns with findings from a study estimating the global and regional burden of infection-attributable cancers using the GLOBOCAN 2018 database. One study reported a greater incidence of HPV infection-attributable cancer in SEA than in East Asia (9.6 vs. 5.8 per 100,000 person-years).^22^ While STI prevention and control efforts in Thailand have effectively reduced the incidence of HIV,^23^^,^^24^ there has been an increase in the incidence of KS caused by this virus among males.^23^ Monitoring infectious diseases, especially STIs, is essential, especially in some at-risk populations.

The incidence of epithelial tumors of the eye and adnexa is significantly higher (95% CI RR > 1) in Thailand when compared to Japan, Korea, and Taiwan as well as the comparison results with Malaysia. These remarkable results confirmed the findings of one study investigating the global eye cancer burden. The incidence of eye cancer was highest in Sub-Saharan Africa (ASR = 4.06 per 100,000 person-years), followed by Western Europe (ASR = 0.89), while a lower incidence was observed in Asian regions. The incidence of this disease was higher in the SEA region than in the East Asia region.^25^ Several studies have shown the effect of ambient ultraviolet radiation on eye cancer.^25^, ^26^, ^27^ This may also explain the greater incidence of epithelial tumors of the eye and adnexa in Thailand than in other regions, as it is likely that the Thai population is more exposed to sunlight than are the other countries participating in this study due to their agricultural careers. The main source of income for the northern Thai population is agriculture.^28^ Another possible factor is bacterial infection. A study in the U.S. population showed that incidence rates of eye cancer were inversely correlated with the percentage of the population receiving fluoridated water. Higher rates were found in states with a lower prevalence of fluoridation.^29^ However, there are several potential factors that cause eye cancer, such as race, eye color, diabetes, obesity, unhealthy diet, smoking, and alcohol consumption.^25^ We encourage further research to determine this phenomenon since the main risk factors for eye cancer in Thailand remain unknown.

The results from our study support that high-income countries continue to have higher childhood cancer incidence and survival rates.^30^^,^^31^ The incidence of childhood cancer and hematological cancer was lower in both Thailand and Malaysia than in Japan, Korea, and Taiwan. A lack of access to diagnostic and imaging facilities as well as a shortage of resources in the healthcare industry may be contributing factors.^32^ The infrastructure supporting childhood cancer care differs between high-income and low-income countries (LMICs), contributing to variations in clinical training and awareness. Consequently, physicians in high-income countries may be more inclined than those in LMICs to conduct early assessments for indications of cancer. Although, five-year survival significantly improved by at least 2% per year from 1990 to 2011 in Songkhla, Southern Thailand for leukemia, acute lymphoblastic leukemia (ALL), and acute myeloid leukemia (AML), the overall 5-year relative survival for leukemia in Songkhla was lower than that reported in the US (43% vs. 79%).^30^

The PBCR in Malaysia and two longstanding registries in Northern Thailand, the Chiang Mai Cancer Registry and the Lampang Cancer Registry, are considered high quality based on a periodic assessment of cancer registries from around the world at IARC, Cancer Incidence in Five Continents (CI5).^33^ The data quality, as indicated by MV% and NOS% is generally satisfy for epithelial tumours of the nasopharynx (94.7% MV and 8.5% NOS for Thailand; 98.9% MV and 25.3% NOS for Malaysia) and for epithelial tumours of the eye and adnexa (88.0% MV and 16.0% NOS for Thailand; 91.3% MV and 23.9% NOS for Malaysia). In contrast, the data for epithelial tumours of the liver and intrahepatic bile tract require cautious interpretation, as the MV% and NOS% are 18.1% and 83.3%, respectively, for Thailand, and 72.6% and 7.0%, respectively, for Malaysia (Supplementary Table S1). The data quality of PBCR in Japan, Korea and Taiwan have been reported by Matsuda et al.^2^ The participating cancer registries report that data completeness is generally achieved within approximately 2–3 years after diagnosis. This lag reflects the time required for case ascertainment and validation processes, which are consistent with international standards for completeness and timeliness.^34^

Population-based cancer registries that capture childhood cancer cases are crucial in LMICs. The high-quality childhood cancer registry can be used to measure incidence and survival. This epidemiology of cancer can help to determine whether childhood cancer is being properly detected, diagnosed, or treated and whether it is necessary to develop interventions and improve diagnosis and prognosis in LMICs.^35^ The need to address issues at the health systems level to improve outcomes in lower resource settings has been well documented.^36^

Several studies have reported the epidemiology of common cancers in Southeast Asia, including Thailand and Malaysia.^37^, ^38^, ^39^ One of the sustainable development goals (SDGs) is to reduce inequality. Although the number of rare cancers is low, these cancers could imply clinical challenges and economic burdens, similar to other rare diseases,^40^ particularly for LMICs. Significant progress has been made in the management of rare diseases and rare cancers in SEA countries. However, there remain important areas for extensive development opportunities.^41^ Since 2018, Malaysia has been actively addressing rare diseases and rare cancers to properly acknowledge them as significant national healthcare concerns.^42^^,^^43^ Central to these efforts is the establishment of a rare disease registry, defining a disease as rare if its prevalence is 1 in 4000 cases per year in Malaysia—a definition supported by the Malaysian Rare Disorders Society. This initiative aims to focus on diseases currently lacking government support, facilitating policy planning and holistic management of rare diseases within the National Framework for Rare Disease in Malaysia. Additionally, the registry seeks to streamline the designation and registration of orphan drugs, promoting harmonization and ensuring a uniform standard nationwide. As of March 2023, the updated Malaysia Rare Disease List (MRDL) includes 44 types of cancer.^44^ However, current prevalence estimates rely on data mainly from developed Western countries and do not reflect the true prevalence in Malaysia. Despite this limitation, this approach is deemed suitable until local Malaysian registries can offer precise prevalence data for each rare disease.

For the first time, we were able to provide detailed statistics on cancer from the RARECAREnet list that can be used as initiative information to plan for cancer control. In our study, for cancer burden, data from the PBCR were used, which can be regarded as an unbiased source of cases. We also examined geographic variance and identified regional, ethnic, and other subgroup differences that may be related to particular environmental or genetic risk factors.^45^

However, our study has several limitations. One limitation is that we could not combine the Thailand and Malaysia data because only the aggregate results were shared and compared. Moreover, the number of participating countries was too small, preventing us from fully representing SEA. Enlarging the participation in RARECARENETAsia to other SEA countries, such as Singapore, Brunei, Indonesia, Vietnam and the Philippines, where PBCRs are available, will provide more precise and accurate estimations of the rare cancer burden in this region. In addition, since quality of both topology and morphology data is essential to classify Tier 2 in the RARECAREnet list, only Tier 1 could be classified in our study, which included the NOS morphologies of any site because the MV% was not sufficient, ranging from 18% to 100% in Thailand and 73%–100% in Malaysia. Another limitation is that the data from Thailand only represent the population in northern Thailand. To enhance comparability with the Malay population in Malaysia, it would be more appropriate to also include PCPR data from southern Thailand. This region shares similarities in population characteristics, culture, and health-related behaviors with Malaysia, where Malays constitute the predominant ethnic group. Therefore, incorporating high-quality cancer data from other regions of Thailand is recommended to provide a more comprehensive estimation of national-level results and to understand geographical variations within the country. On the other hand, the PBCR in Malaysia faces challenges in obtaining notifications of cancer cases from private health facilities. Despite this obstacle, the registry compensates for active case search collaborations, estimating its data completeness to be over 70 percent.

Furthermore, the evaluation of incidence trends for rare cancers was constrained by the limited availability of high-quality longitudinal data. Comprehensive analysis over a ten-year period is essential to accurately detect increases in rare cancer incidence and to inform the prioritization of public health interventions. Sugiyama et al.^46^ reported the trend in incidence of rare cancers in Japan and found that the ASR for epithelial tumors of gallbladder decreased and epithelial tumors of stomach and liver decreased. This has not yet been documented in SEA. Due to limited data quality for specific cancer sites in SEA, conducting analyses comparable to those performed for the Japanese population remains challenging. Our findings provide a foundation for analyzing incidence trends of specific cancers, particularly infection-related rare cancers that are more prevalent in Thailand and Malaysia than in Japan, Korea, and Taiwan.

In conclusion, while many rare cancers identified by RARECAREnet are indeed rare in Thailand and Malaysia, some cancers categorized as rare in Europe do not meet the rarity criteria in this region. Additionally, according to RARECAREnet list, certain cancers that are rare in Thailand and Malaysia had higher ASRs in Japan, Korea, and Taiwan. To improve the diagnosis and understanding of rare cancers, robust epidemiological data must be developed through initiatives such as the RARECAREnet Asia project. This involves enhancing the quality of cancer registry, especially for hematological and childhood cancers, and collaborating with potential PBCRs across Asia.

## Contributors

PS, SY, and TM contributed equally to this work. PS, SY and TM contributed to the conception and design, data acquisition, interpreted results and drafted the manuscript. DP, IC, BB, NSI, KD, NW, YW, and RCC contributed to data acquisition and interpretation of results. PS, BB, and HC performed the statistical analysis. KS and AT contributed to the conception and interpretation of results. All authors reviewed and approved the final manuscript.

## Data sharing statement

The datasets used and/or analyzed during the current study are available from the corresponding author upon reasonable request.

## Declaration of interests

The authors declare that they have no competing interests.
